# Triple HIV-1 Infection Is Associated With Faster CD4^+^ T-Cell Decline

**DOI:** 10.3389/fmicb.2020.00021

**Published:** 2020-01-24

**Authors:** Yu Zhang, Bin Su, Hanping Li, Jingwan Han, Tong Zhang, Tianyi Li, Hao Wu, Xiaolin Wang, Jingyun Li, Yongjian Liu, Lin Li

**Affiliations:** ^1^Department of AIDS Research, State Key Laboratory of Pathogen and Biosecurity, Beijing Institute of Microbiology and Epidemiology, Beijing, China; ^2^Center for Infectious Diseases, Beijing Key Laboratory for HIV/AIDS Research, Beijing Youan Hospital, Capital Medical University, Beijing, China

**Keywords:** HIV, diversity, dual infection, triple infection, disease progression

## Abstract

HIV-1 dual infection occurs when an individual is simultaneously or sequentially infected with two or more genetically distinct HIV-1 strains. According to the number of infected strains, HIV-1 dual infection can be divided in double infection and triple infection and so on. Currently, the majority of dual infection cases have been reported to be double infections which can result in detrimental clinical outcomes. The high incidence of double infection among specific high-risk populations increases the likelihood of triple infection, which has been sporadically described. There is no doubt that we are concerned about the association between triple infection and disease progression. However, this relationship is still unclear on the population level. In this study, 70 individuals from the Beijing PRIMO cohort were longitudinally followed up with a median time of 15.75 months for the purpose of investigating the incidence of dual infection. Phylogenetic analyses using bulk and single-genome sequences showed that nine individuals acquired double infection, with the incidence of 9.21 per 100 person-years, and three individuals with triple infection were identified, with the incidence of 3.07 per 100 person-years. The further survival analysis demonstrated that the triple infection group exhibited faster CD4^+^ T-cell decline. In summary, these results demonstrate for the first time that the triple HIV-1 infection might reduce CD4^+^ T-cell counts, which would predict a more rapid disease progression.

## Introduction

Human immunodeficiency virus type 1 (HIV-1) exhibits high genetic diversity and a formidable pattern of evolution (McCutchan et al., 1996), which is driven in large part by the high mutation and recombination rates of the reverse transcriptase enzyme. Currently, there are 91 reported circulating recombinant forms (CRFs), and numerous unique recombinant forms (URFs) have been identified^1^. The emerging CRFs and URFs are the best circumstantial evidence for dual infection, which is defined as infection of the same individual with two or more genetically distinct strains. Depending on the timing of infection with the second strain, dual infection can be subdivided into two groups: co-infection is defined as infection with two heterologous strains either simultaneously or within a brief period of time before seroconversion, while superinfection is defined as the infection with a second strain after establishment of an immune response to the initial infection (Smith et al., 2005).

The issue of dual infection has drawn considerable critical attention due to the detrimental effects on clinical outcomes. Studies have determined that dual infection is related to higher viral loads and faster CD4^+^ T-cell decline, and the acquisition of a superinfected drug-resistant strain could directly lead to the failure of antiretroviral therapy (ART) (Gottlieb et al., 2004; Cornelissen et al., 2012; Pingen et al., 2012). In previous studies, almost all dual infection cases had double infection, indicating that infection with more than two HIV-1 strains seemed not to occur due to the protective immunity induced by double infection (Basu et al., 2012; Cortez et al., 2012). However, multiple HIV-1 infection is not impossible considering the high incidence of double infection in some regions (Kraft et al., 2012; Redd et al., 2014; Wagner et al., 2014). To date, only a few cases of triple HIV-1 infection have been reported (Takehisa et al., 1997; Gerhardt et al., 2005; van der Kuyl et al., 2005; Pernas et al., 2006; de Azevedo et al., 2019).

One of these cases of triple infection was reported in a 35-year-old homosexual man who did not have clinical symptoms and who did not have a reduced CD4^+^ T-cell count when he was infected with two distinct subtype B strains, whereas infection of a third divergent CRF01_AE strain resulted in a high viral load with a markedly reduced CD4^+^ T-cell count and acute viral illness (van der Kuyl et al., 2005). Unlike this case, another individual, who was an elite controller, only displayed a transient peak of viremia when the acquisition of a third intersubtype strain occurred and presented no evidence of immunologic progression for at least 2 years after the triple infection event (de Azevedo et al., 2019). Based on the sporadic reported cases, the association between triple HIV-1 infection and disease progression is still uncertain.

Men who have sex with men (MSM) have always been associated with high-risk sexual behaviors (multiple sexual partners and unprotected sexual intercourse), which might increase the opportunities for dual infection (Zhang and Chu, 2005; Shang et al., 2012). A previous investigation revealed that the incidence of double infection was up to 19.6 per 100 person-years among MSM in Liaoning Province of China, which is much higher than most regions in the world (Luan et al., 2017). In addition, the identification of numerous new CRFs and URFs (Han et al., 2013a, b; Feng et al., 2014; Li et al., 2017; Zhang et al., 2019) and confirmation of onward transmission of URFs among MSM in China (Liu et al., 2019) provided indirect evidence indicating that it was possible to identify cases of triple infection among Chinese MSM. To this end, 70 participants from the Beijing PRIMO cohort were enrolled in this study, and 3 participants were identified to have triple infection. Therefore, we reported for the first time the incidence of triple infection and evaluated the effect of triple infection on disease progression.

## Materials and Methods

### Study Population

The Beijing PRIMO cohort, started in October 2006 in the HIV care clinic at Youan Hospital, was an opening prospective study cohort of HIV-1 seronegative MSM (described in detailed in Huang et al., 2013). After seroconversion, clinical and laboratory measurements were taken at weeks 1, 2, 4, 8, and 12 and then every 3 months. The estimated date of infection (EDI) was defined as the midpoint between the last negative HIV-1 antibody test and the first HIV-1 antibody positive test or as 14 days prior to a positive RNA PCR assay on the same day as a negative HIV Enzyme Immunoassay. Acute/early HIV-1 infection was defined as (a) a positive HIV-1 RNA with a negative or indeterminate HIV-1 antibody test, followed by HIV seroconversion within 6 months; or (b) a negative ELISA and Western blot (WB) less than 180 days before a documented positive ELISA or WB. Individuals who were ART naive, had deferred ART for at least 6 months after their EDI, and had at least two blood plasma samples (one sample of acute/early infection) available for sequencing were included in this study for the purpose of investigating the occurrence of dual infection. The opportunistic infections, tuberculosis, autoimmune diseases or HBV/HCV co-infection were excluded for all study participants.

### Bulk *gp41* and *p24* Gene Amplification and Sequencing

As *gp41* (401 bp) and *p24* (432 bp) coding regions showed structural stability and relatively limited intrahost evolution, they were chosen to screen for dual infection (Redd et al., 2014; Zhang et al., 2017). A total of 200 μl of plasma was collected at each follow-up time point from each patient and centrifuged at 24000 *g* for 1 h at 4°C; 150 μl of supernatant was stored at −80°C, and the remaining 50 μl of precipitate was used for viral RNA isolation according to the manufacturer’s instructions for the High Pure Viral RNA Kit (Roche, Germany). Nested PCR amplification for *gp41* and *p24* was performed using the One Step RNA PCR Kit (Takara, RR055A) and the Premix ExTaq Kit (Takara, RR902A) with same thermal cycling conditions but different sets of primers as described in Table 1. The final PCR products were analyzed by 1% agarose gel electrophoresis, and the target-sized products were purified and directly subjected to Sanger sequencing.

**TABLE 1:** Primers used for PCR amplification.

Primer names	Direction	Primer sequence	Positions in HXB_2_
**gp41**			
**Outer primers**			
gp41_F1	Forward	5′-TCTTAGGAGCAGCAGGAAGCACTATGGG-3′	7789–7816
gp41_R1	Reverse	5′-AACGACAAAGGTGAGTATCCCTGCCTAA-3′	8347–8371
**Inner primers**			
gp41_F2	Forward	5′-ACAATTATTGTCTGGTATAGTGCAACAGCA-3′	7850–7879
gP41_R2	Reverse	5′-TTAAACCTATCAAGCCTCCTACTATCATTA-3′	8281–8310
**p24**			
**Outer primers**			
p24_F1	Forward	5′-TCACCTAGAACTTTAAATGCATGGG-3′	1231–1255
p24_R1	Reverse	5′-CACTCCCTGACATGCTGTCATCAT-3′	1825–1848
**Inner primers**			
p24_F2	Forward	5′-TAGCCCAGAAGTAATACCCATGTT-3′	1281–1304
p24_R2	Reverse	5′-TGGACTAGCAAGGTTTCTGTCATC-3′	1737–1760

### Bulk PCR Sequence Analyses

Pre-selection of individuals with dual infection was performed by counting the degenerate bases (DB) in bulk sequence (van der Kuyl and Cornelissen, 2007), and dual infection could also be shown by the divergence of follow-up sequences, which were measured by the maximum pairwise genetic distance (PD) and visualized by Highlighter tool (Kraft et al., 2012).

For this purpose, bulk PCR sequences were manually assembled in Contig Express software, and the nucleotide positions where the secondary peak was at least 30% as high as the primary peak were counted as the DB using the International Union of Pure and Applied Chemistry (IUPAC) designations. Longitudinal sequences (*gp41 and p24*) from each individual were aligned using the ClustalW multiple alignment programs included in BioEdit software, and PD was calculated based on the Tamura-Nei genetic distance model with 1000 bootstrap replicates in MEGA 6.0 software. A scatter diagram was plotted with the maximum PD as the *X*-axis and the maximum percentage of DB as the *Y*-axis. Individuals who had a higher percentage of either parameter or both than the majority individuals clustered in the low percentage were inferred to have dual infection.

In addition, the Highlighter tool from the Los Alamos National Laboratory (LANL) HIV Sequence Database^2^ was used to map mutations and DB deviating from the earliest sample. The heterogeneity of the viral population in individuals with dual infection could be visualized at each follow-up time point relative to the acute/early infection time point. For individuals who had dual infection in both analyses, single-genome amplification (SGA) of *gp41* or *p24* was further performed.

### Single-Genome Amplification and Sequencing of *gp41*

As described in the Supplementary Methods, viral RNA extracted from the inferred dually infected individuals was reverse-transcribed to *gp41* complementary DNA (cDNA) using SuperScript^®^ IV First-Strand Synthesis System (Invitrogen, Catalog No: 18080-051), and cDNA was endpoint diluted in 96-well plates such that fewer than 29 PCRs yielded a amplification product. According to a Poisson distribution, the cDNA dilution that yields PCR products in no more than 30% of wells contains one amplifiable cDNA template per positive PCR more than 80% of the time (Salazar-Gonzalez et al., 2008). The diluted *gp41* cDNA was used to perform first-round PCR according to the protocol of the ExTaq DNA Polymerase Kit (Takara, RR001B), and the second-round amplification was carried out based on the same conditions as the bulk PCR amplification using the Premix ExTaq Kit (Takara, RR902A). All products derived from cDNA dilutions yielding less than 30% PCR positivity were sequenced.

### Phylogenetic Analyses and Definition of Dual Infection

In the phylogenetic analyses, we included a reference data set containing 15 sequences with 5 sequences of subtype B, CRF01_AE and CRF07_BC identified in our previous study (Liu et al., 2019). To eliminate cross-contamination between samples and contamination from the laboratory, all SGA sequences were input into BLAST against a dataset of locally generated SGA sequences and laboratory-developed molecular clones. Longitudinal SGA sequences from a putative individual with dual infection were aligned with the reference sequence dataset. A neighbour-joining (NJ) phylogenetic tree was constructed in MEGA6.0 using the Kimura 2-parameter model with 1000 bootstrap replicates to access evidence of dual infection. The individuals were classified as double or triple infection if 2 or 3 clusters were separated by branches of reference sequences with a bootstrap value > 80%. In addition, PD between any two clusters should exceeded 2.5% for the following reasons: (a) the maximum within-patient diversity that could plausibly develop from a single infecting virus within 100 days was 0.6%; (b) the average follow-up of each individual in this study was approximately 400 days (Salazar-Gonzalez et al., 2008). Co-infection was defined as dual infection at baseline, and superinfection was defined as single infection at baseline and dual infection at a later time point.

### Statistical Analyses

All individuals were divided into groups with single, double and triple infections. Baseline CD4^+^ T-cell counts were compared between the three groups using the Mann–Whitney test, and the longitudinal slopes of CD4^+^ T-cell counts calculated by linear regression were also compared. Disease progression was defined as the time between seroconversion and the end point (CD4^+^ T-cell counts ≤ 350 cells/μl). We measured the rate of disease progression across the groups using Kaplan–Meier plots and log-rank tests. All statistical analyses were performed based on a two-sided α of 0.05 using R version 3.5.1.

## Results

### Study Population

Among the individuals participating in the Beijing PRIMO cohort between March 2010 and November 2011, 70 individuals meeting the inclusion criteria were enrolled in this study. Follow-up was 97.69 person-years in total and 15.75 months (IQR: 13.58–20.50 months) on average; 10 individuals (14.29%) were followed 0.5–1 year, 51 individuals (71.83%) were followed 1–2 years, and the remaining nine individuals (12.86%) were followed > 2 years. Each individual had a median of four time point plasma samples for sequencing. At baseline, the median time was 50 days (IQR: 40.00–65.75 days) from EDI. Additional demographic information is summarized in Table 2.

**TABLE 2:** Demographic information of study participants.

Group	*N* (%)
**Age**	
<20	2 (2.9)
20–39	56 (80.0)
40–59	9 (12.9)
>59	1 (1.4)
Unknown	2 (2.9)
**Marriage**	
Unmarried	46 (65.7)
Married	14 (20.0)
Divorced or Single	9 (12.9)
Unknown	1 (1.4)
**Nationality**	
Han	64 (91.4)
Minority	5 (7.1)
Unknown	1 (1.4)

### Screening for Dual Infection Using Bulk *gp41* and *p24* Sequences

**FIGURE 1:**
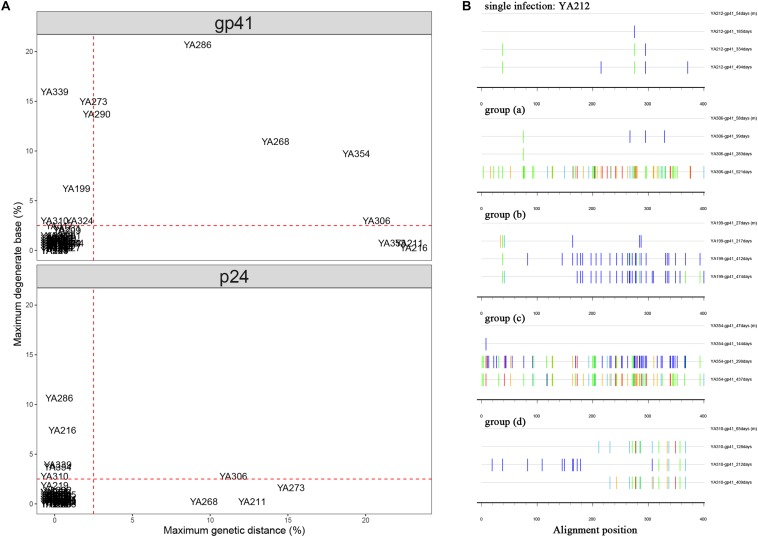
**(A)** Comparison of maximum pairwise genetic distance (PD) and maximum percentage of degenerate bases (DB) using longitudinal bulk gp41 and p24 sequences from each individual. The maximum PD is plotted on the *X*-axis, and the maximum percentage of DB is plotted on the *Y*-axis. Individuals who had the parameters of PD or DB higher than the cut-off (red dashed lines) are inferred to have dual infection. **(B)** Highlighter plots for the representatives of single infection and inferred dual infection are shown. The sequence at each follow-up time point is compared to the baseline sequence, and the name marked on the right of each sequence is structured with the patient name-sequence region-estimated date of infection (EDI). Tick marks denote nucleotide changes from the baseline sequence (T, red; A, green; C, light blue; G, orange; DB, dark blue).

For each individual, a combination of *gp41* and *p24* bulk sequences from each time point plasma sample was used to screen for dual infection. In the analysis using bulk *gp41* sequences, a majority of individuals (56/70, 80%) clustered in the low percentage (<2.5% PD, <2.5% DB), which were considered as single infections. The other 14 individuals were generally divided into the following four groups: (a) four individuals exhibited a high percentage of PD (>5%); (b) four individuals exhibited a high percentage of DB (>5%); (c) three individuals exhibited a high percentage of both parameters (>5% PD, >5% DB); (d) three individuals (YA310, YA317, and YA324) exhibited a relatively high percentage of DB (2.5–5%) but a low percentage of PD (<2.5%). To avoid missing cases of dual infection, we performed the same analysis using *p24* sequences. The results did not reveal additional individuals with evidence of dual infection, while three individuals (YA199, YA290, and YA353) who had a high percentage of PD or DB in the previous analysis of *gp41* sequences clustered in the low percentage (Figure 1A).

The Highlighter plot, which provided a visual representation of homogeneity or heterogeneity of the viral population at each time point relative to the virus population at baseline, was also a useful tool to predict dual infection. The nucleotides that do not match the baseline sequence were assigned a color unique to each nucleotide (A = green, T = red, G = orange, C = light blue, degenerate = dark blue). This analysis demonstrated that the *gp41* sequences remained relatively homogeneous with no evidence of dual infection for most individuals (56/70, 80%), which is consistent with the result shown in the scatter diagram. A representative plot of YA212 exhibited minimal changes over 494 days after EDI in the longitudinal gp41 sequences (1 change at 185 days, 3 changes at 334 days and 5 changes at 494 days). The representative Highlighter plots of the other 14 individuals from the above four groups were shown in Figure 1B.

In summary, 11 individuals were identified with dual infection through *gp41* and *p24* bulk sequence analyses, but we also could not exclude the possibility that three individuals with a relatively high percentage of DB but low PD were dually infected. Therefore, all 14 of these individuals underwent SGA to confirm dual infection.

### Confirmation of Dual Infection by SGA

In the process of SGA-derived sequence analysis, we obtained enough sequences from each time point sample of 13 individuals except for YA211 (Table 3). We only collected the SGA-derived sequences at 214 and 741 days (after EDI) from YA211, while the samples from 46, 96, and 376 days (after EDI) failed to amplify due to low viral load. In the phylogenetic analyses, YA211 had double infection with subtype B strains identified at 214 days (after EDI) and CRF01_AE identified at 741 days (after EDI). However, we could not determine whether YA211 was co-infected or superinfected. For the other 13 individuals, we identified 8 double infections (2 co-infections, 6 superinfections) and 3 triple infections (1 co-infection, 2 superinfections).

**TABLE 3:** Determination of dual infection by SGA-derived sequences.

Subject ID	Time point (days after EDI)/Number of gp41 SGA sequences					HIV-1 subtype					Type of dual infection
			
	1	2	3	4	5	1	2	3	4	5	
YA199	27/22	217/22	412/20	474/18	–	01AE	01AE/01AE	01AE/01AE/07BC	01AE/01AE	–	Superinfection (Triple)
YA211	46/–	96/–	214/16	376/−	741/27	–	–	B	–	01AE	Cannot determined (Double)
YA216	66/27	111/29	235/26	458/27	805/21	07BC	07BC	07BC	07BC	01AE	Superinfection (Double)
YA268	46/30	74/13	213/20	412/14	–	07BC	07BC	07BC/B	B	–	Superinfection (Double)
YA273	47/23	123/26	222/33	419/36	–	07BC	07BC	07BC	07BC/01AE	–	Superinfection (Double)
YA286	30/31	197/47	365/41	–	–	01AE/B	01AE/B	01AE/B	–	–	Co-infection (Double)
YA290	65/28	109/23	219/34	–	–	01AE/07BC	01AE/07BC	01AE/07BC	–	–	Co-infection (Double)
YA306	58/27	99/23	283/30	521/34	–	01AE	01AE	01AE	07BC		Superinfection (Double)
YA310	65/22	128/9	212/33	409/30	–	01AE/01AE/01AE	01AE	01AE/01AE	01AE	–	Co-infection (Triple)
YA339	38/36	113/40	212/28	386/18	–	01AE	01AE/07BC	01AE/07BC/07BC	01AE	–	Superinfection (Triple)
YA353	68/44	217/36	473/18	–	–	B	B	B/01AE	01AE	–	Superinfection (Double)
YA354	47/34	144/35	299/21	437/25	–	01AE	01AE	01AE/07BC	01AE/07BC	–	Superinfection (Double)

*EDI, estimated date of infection; SGA, single-genome amplification.*

**FIGURE 2:**
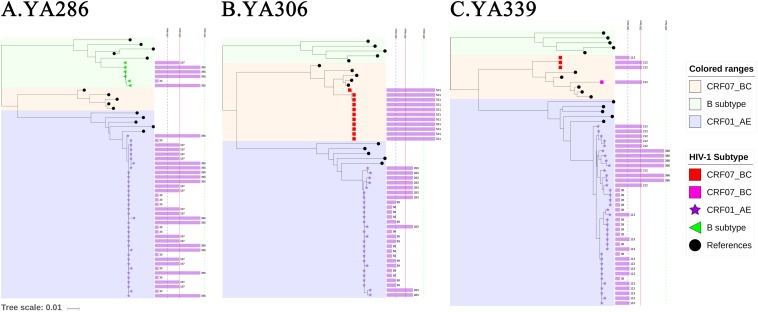
The neighbour-joining (NJ) phylogenetic trees were generated using longitudinal *gp41* (401 bp) SGA-derived sequences extracted in one-third proportion. YA290, YA306 and YA339 are examples illustrating **(A)** double infection (co-infection), **(B)** double infection (superinfection), and **(C)** triple infection (superinfection). Colored ranges indicate the branches of different subtypes (B subtype: light green, CRF01_AE: light blue, CRF07_BC: light pink). SGA-derived sequences from patients are also represented by different shapes (B subtype: green left triangle, CRF01_AE: purple star, CRF07_BC: red or pink square), and solid black circle indicates references. The purple bar shown on the right of the phylogenetic trees indicates the sampled time after the EDI, and the values are labeled.

The representative NJ phylogenetic trees of individuals who were co-infected, superinfected and triple-infected are shown in Figure 2. YA306, the representative of group (a), was an individual with superinfection exhibiting a high percentage of PD. In the phylogenetic analysis, YA306 was initially infected with CRF01_AE until 521 days after EDI when the viral populations were replaced by CRF07_BC. To represent group B, the phylogenetic tree of triple-superinfected individuals (YA339) is shown in Figure 2B. YA339 was infected with CRF01_AE strains over all 386 days; however, there were additional CRF07_BC strains at 113 and 212 days (after EDI), and four sequences of CRF07_BC at 212 days (after EDI) formed a genetically distant branch that was approximately 5.1% divergent from the CRF07_BC at 113 days, indicating that YA339 was a triple-infected subject. Another example is YA286 from group (c), who was co-infected. This individual was initially infected by two variants (CRF01_AE and B subtype), evidenced by two distinct branches in the NJ phylogenetic tree. Although the dominant strain was CRF01_AE for the duration of follow-up, we identified that the proportion of B subtype strains also increased from baseline to 365 days (after EDI).

**FIGURE 3:**
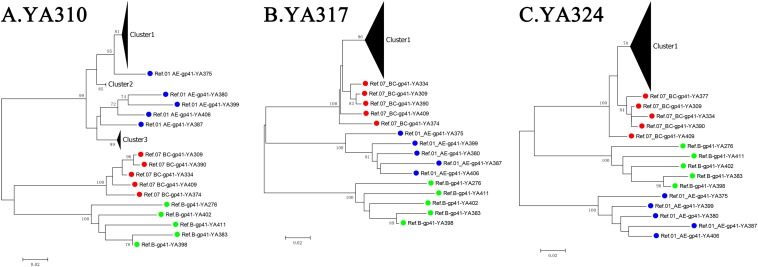
The neighbour-joining (NJ) phylogenetic trees were generated using longitudinal *gp41* (401 bp) SGA-derived sequences of three individuals from group (d): YA310 **(A)**, YA317 **(B)**, and YA324 **(C)**. Reference sequences are labeled as circles with different colors (B subtype = green, CRF01_AE = blue, CRF07_BC = red).

For the three individuals in group (d), YA310 had a triple co-infection and was initially infected with CRF01_AE, forming three distant clusters (the PD between any two clusters > 2.5%) at baseline, which were separated by the branches of the reference sequence. For YA317 and YA324, the within-patient SGA-derived sequences exhibited divergence but with low PD (<2.5%), and they were not separated by the branches of reference sequences (Figure 3). As a result, these two individuals were classified as single infection.

### High Prevalence and Incidence of Double Infection and Triple Infection

In this study, a total of 12 dual infections were identified, and the cumulative incidence and prevalence were 12.28 per 100 person-years and 17.14%, respectively. Three co-infections and eight superinfections were identified by dividing by the timing of infection with the second strain, while the other strain could not be determined because of unknown baseline infection. The incidence and prevalence of superinfection were 8.19 per 100 person-years and 11.43%, respectively, and for co-infection, the prevalence reached 4.29%. Nine double infections and three triple infections were identified. The incidence and prevalence of double infection was up to 9.21 per 100 person-years and 12.86%, respectively. Notably, this is first report identifying the incidence and prevalence of triple infection, which were 3.07 per 100 person-years and 4.29%, respectively.

### Triple Infection Is Associated With Disease Progression

**FIGURE 4:**
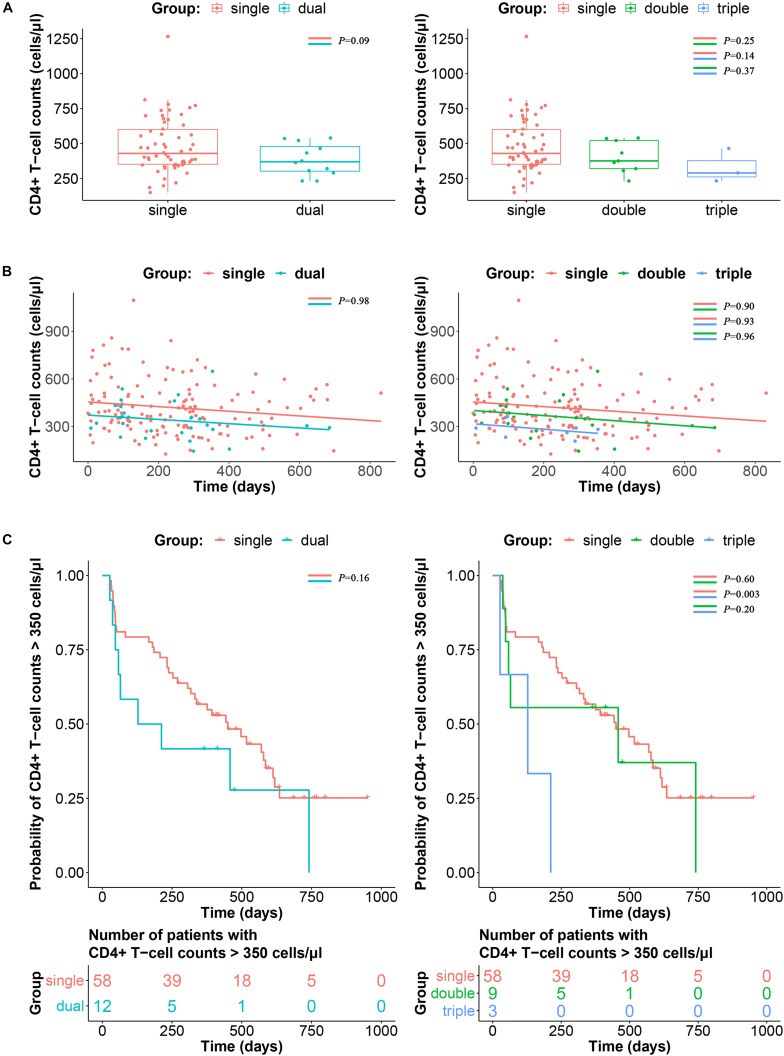
Impacts of triple infection on disease progression. **(A)** CD4^+^ T-cell counts at adjusted baseline are compared between single and dual infection (left panel) or single, double and triple infection participants (right panel). *P*-values are calculated using the Wilcoxon rank sum test or Kruskal–Wallis *H* test. **(B)** Longitudinal slopes after adjusted baseline and the time-weighted change from the adjusted baseline of CD4^+^ T-cell counts were calculated by linear regression, and then compared between single and dual infection (left panel) or single, double and triple infection participants (right panel). **(C)** Kaplan–Meier curve of the time to reach CD4^+^ T-cell counts ≤ 350 cells/μl according to single and dual infection (left panel) or single, double and triple infection participants (right panel). *P*-values were calculated using the log-rank test.

To avoid the impact of viral load fluctuation on the CD4^+^ T-cell counts at the early stage of HIV-1 infection, data from 120 days after EDI were used as baseline for comparison (Luan et al., 2017). As shown in Figure 4A, the CD4^+^ T-cell counts at baseline were not significantly different between single infection, double infection and triple infection (median 429.5, 376 versus 290 cells/μl; *P* > 0.05). Similarly, longitudinal slopes after adjusted baseline and the time-weighted change from the adjusted baseline of CD4^+^ T-cell counts were also not significantly different between these three groups (*P* > 0.05) (Figure 4B).

In the survival analysis to determine the impact of triple infection on disease progression, time to the achievement of CD4^+^ T-cell counts ≤ 350 cells/μl was significantly shorter in the triple infection group than in the single and double infection groups (*P* < 0.05). However, no significant difference was detected between the double and single infection groups (*P* > 0.05) (Figure 4C).

## Discussion

Accurate detection of dual infection is the basis for further research, yet it is complex. In previous studies, bulk viral sequence analysis, SGA and next-generation sequencing assays (NGS) are the three diagnostic strategies that are widely used for detection (Jurriaans et al., 2008; Sheward et al., 2015; Hebberecht et al., 2018; Popescu et al., 2018). HIV-1 reverse transcriptase (RT) bulk sequences are routinely available in clinical practice, and a high number of DB in RT indicates the possibility of a dual infection (Cornelissen et al., 2007). Not surprisingly, this method poorly detected minor variants lower than 30% (Rachinger et al., 2010). Although high sensitivity of detecting dual infection can be achieved by NGS (Redd et al., 2011), it is subject to experimental biases, especially preceded by PCR amplification. Among these three strategies, SGA can be the best way to obtain viral quasispecies sequences, but this approach is prohibitively expensive and too labor-intensive for large-scale studies (Salazar-Gonzalez et al., 2008). Therefore, using bulk sequences to screen for dual infections can reduce experimental cost; in addition, confirmation by SGA increases the accuracy of detection in this study.

Based on this diagnosis strategy, nine double infections were identified, and the cumulative incidence of double infection was 9.21 per 100 person-years, which was lower than the result reported by the previous survey conducted in Liaoning Province (Luan et al., 2017). With three additional triple infections identified in this study, the incidence of triple infection of 3.07 per 100 person-years among MSM in China was first reported. Of these 12 individuals, 11 had intersubtype HIV-1 double or triple infections, and only one had an intrasubtype triple infection. These 11 individuals with intersubtype double or triple infection confirmed by SGA-derived sequence analyses are consistent with individuals who had a high percentage of either parameter of both (PD > 5%, DB > 5%) in bulk *gp41* sequence analyses. However, for the individuals who had relatively high DB or PD parameters (2.5–5%), determining whether they had dual infection was not easy. Three individuals exhibited a relatively high DB parameter (2.5–5%) but a low PD percentage (<2.5%). Finally, only YA310 was confirmed to be intrasubtype triple infection, while YA317 and YA324 were more likely infected with multiple intrasubtype founder viruses. We can conclude from the results that bulk sequence analyses were sensitive for screening for intersubtype infection but not intrasubtype dual infection. Specifically, a certain proportion of multivariant transmission complicated the identification of dual infection. To distinguish dual infection from multivariant transmission, phylogenetic analysis using SGA-derived sequences is a better choice (Li et al., 2010).

According to previous studies, approximately 20–35% of individuals become infected with multiple founder HIV-1 variants (Keele et al., 2008; Abrahams et al., 2009). The association between the diversity of founder viruses and higher viral loads was supported by a previous report (Janes et al., 2015). Therefore, it would be natural to assume that sequential infection with multiple HIV-1 strains could also influence disease progression. In the survival analysis, triple infection showed more faster CD4^+^ T-cell decline than double and single infection (*P* < 0.05). However, double infection had a limited impact on the decline in CD4^+^ T-cell counts than single infection (*P* > 0.05), which is consistent with the results of the study conducted on female sex workers in Kenya (Ronen et al., 2014), but contradict findings obtained from the cohort of men with primary HIV infection (Cornelissen et al., 2012). It was possibly because of the shorter follow-up time of these individuals from the cohort, as individuals with double infection initiated ART at higher CD4^+^ T-cell counts. There was no significant difference in CD4^+^ T-cell counts among single, double and triple infection at baseline (*P* > 0.05, Figure 4A), whereas CD4^+^ T-cell count appeared to trend lower in the triple infection group compared to the single infection group, although it was not statistically significant (*P* = 0.14, Figure 4A). Therefore, although longitudinal slopes showed no difference between patient groups (Figure 4B), it is likely that the faster CD4^+^ T-cell decline in triple infected patients is due to lower baseline CD4^+^ T-cell counts. Additionally, the effect of dual infection on rapid disease progression was also reported to be confounded by CCR5-Δ32 heterozygosity and presence of an HLA-B allele (Cornelissen et al., 2012). However, the causality between triple infection and disease progression needs to be further confirmed based on more triple infection cases with comprehensive clinical information.

In addition, this study compared the change in CD4^+^ T-cell counts but could not compare the difference in viral load due to incomplete data recording. The current study is also limited by the relatively short follow-up due to the initiation of ART, thus we may miss some cases of double or triple infection. Another limitation of the study is that the statistical analyses were based on a relatively small number of triple infected patients due to the scarcity of such infections. Despite these limitations, this study is the first to describe the incidence of triple infection among MSM in China and demonstrated that triple infection is associated with faster CD4^+^ T-cell decline, which would predict a more rapid disease progression. Considering the high frequency and incidence of double or triple infection, greater efforts are needed to provide tailored interventions to this population subgroup.

## Data Availability Statement

The gene sequences were deposited in GenBank with accession numbers MN429327 and MN430724.

## Ethics Statement

This study and all the relevant experiments were approved by the Ethics Committees of the Beijing Institute of Microbiology and Epidemiology and Beijing Youan Hospital. All study participants provided written informed consent for the collection of blood samples and subsequent analyses. The methods used conformed to approved guidelines and regulations.

## Author Contributions

YL and LL designed the study. YZ and YL performed the experiments. YZ and XW participated in the process of sequences editing and phylogenetic analyses. HL, JH, and TL performed the statistical analysis. BS, TZ, and HW collected the sample and the demographic data. YZ, BS, JL, YL, and LL participated in the writing process. All authors read and approved the final manuscript.

## Conflict of Interest

The authors declare that the research was conducted in the absence of any commercial or financial relationships that could be construed as a potential conflict of interest.

## Footnotes

1 https://www.hiv.lanl.gov

2 https://www.hiv.lanl.gov/content/sequence/HIGHLIGHT/highlighter_top.html
