# Supplementary material for: Triple HIV-1 Infection Is Associated With Faster CD4+ T-Cell Decline
Source: Front Microbiol. 2020 Jan 24;11:21. doi: 10.3389/fmicb.2020.00021 (PMC6992562; doi:10.3389/fmicb.2020.00021)
Supplement: Supplementary file 1 [file Data_Sheet_1.docx]

**Supplemental Methods**

**One-step RT-PCR reaction (****Takara, RR055A)**

The one-step RT-PCR reaction was performed in a volume of 25 μl as follows: 5 μl RNA, 5.5 μl RNase Free dH_2_O, 12.5 μl 2 X 1 Step Buffer, 1 μl PrimeScript 1 Step Enzyme Mix and 1 μl Primers (0.5 μl Primer_F1 and 0.5 μl Primer_R1). The following PCR conditions were used: 50°C for 30 min, 94°C for 2 min followed by 32 cycles of 94°C for 30 s, 55°C for 30 s and 72°C for 1 min, with a final extension of 72°C for 10 min.

**Second-round PCR reaction (****Takara, RR902A)**

Second-round PCR was carried out in a volume of 30 μl as follows: 1 μl first-round PCR product, 12.8 μl RNase Free dH_2_O, 15 μl Premix Taq and 1.2 μl Primers (0.6 μl Primer_F2 and 0.6 μl Primer_R2). The following PCR conditions were used: 98°C for 2 min followed by 32 cycles of 98°C for 10 s, 55°C for 30 s and 72°C for 1 min, with a final extension of 72°C for 10 min.

**cDNA synthesis (Invitrogen, Catalog No: 18080-051)**

In the first step of reverse transcription (RT), the mixture of 1 μl dNTP Mix (10 mM), 0.1 μl outer reverse primer of *gp41* and 11.9 μl RNA was heated for 5 min at 65°C and incubated on ice for at least 1 min immediately. The annealed RNA was combined with RT reaction mix containing 4 μl 5X SSIV Buffer, 1 μl 100 mM DTT, 1 μl Ribonuclease Inhibitor and 1 μl SuperScript^TM^ Ⅳ Reverse Transcriptase. The combined reaction mixture was incubated at 50°C for 10 min and 80°C for 10 min to synthetize cDNA, and finally it was added with 1 μl RNase H and incubated 37°C for 20 min to remove RNA.

**Fir****st-round single genome amplification (Takara, RR001B)**

First-round PCR was carried out in a volume of 20 μl as follows: 14.9 μl RNase Free dH_2_O, 2 μl 10X Buffer, 1.6 μl dNTP Mixture, 0.4μl primers (0.2 μl gp41_F1 and 0.2 μl gp41_R1), 0.1 μl Takara Ex Taq and 1μl diluted cDNA yielding less than 30% PCR positivity. The following PCR conditions were used: 98°C for 2 min followed by 32 cycles of 98°C for 10 s, 55°C for 30 s and 72°C for 1 min, with a final extension of 72°C for 10 min.
